# Shaping anesthetic techniques to reduce post-operative delirium (SHARP) study: a protocol for a prospective pragmatic randomized controlled trial to evaluate spinal anesthesia with targeted sedation compared with general anesthesia in older adults undergoing lumbar spine fusion surgery

**DOI:** 10.1186/s12871-019-0867-7

**Published:** 2019-10-27

**Authors:** Charles H. Brown, Emily L. Jones, Charles Lin, Melody Esmaili, Yara Gorashi, Richard A. Skelton, Daniel Kaganov, Elizabeth A. Colantuoni, Lisa R. Yanek, Karin J. Neufeld, Vidyulata Kamath, Frederick E. Sieber, Clayton L. Dean, Charles C. Edwards, Charles W. Hogue

**Affiliations:** 1 Department of Anesthesiology & Critical Care Medicine, Johns Hopkins University School of Medicine, Zayed 6208, 1800 Orleans St, Baltimore, MD 21287 USA; 2 Mercy Anesthesiology Associates, 300 St. Paul Place, Baltimore, MD 21202 USA; 3 Tufts University School of Medicine, 145 Harrison Ave, Boston, MA 02111 USA; 4 University of Miami Miller School of Medicine, 1600 NW 10th avenue, Miami, FL 33136 USA; 5 Department of Biostatistics, Johns Hopkins Bloomberg School of Public Health, 615 N. Wolfe St, Baltimore, MD 21287 USA; 6 Department of Medicine, Johns Hopkins University School of Medicine, 1830 Building; 8024, 600 N. Wolfe St, Baltimore, MD 21287 USA; 7 Department of Psychiatry and Behavioral Sciences, Johns Hopkins University School of Medicine, A4 Center Suite 457, 4940 Eastern Avenue, Baltimore, MD 21224 USA; 8 The Maryland Spine Center at Mercy, 301 St. Paul Place, Baltimore, MD 21202 USA; 9 Department of Anesthesiology, Northwestern Feinberg School of Medicine, NMH/Feinberg Room 5-704, 251 E Huron, Northwestern Feinberg School of Medicine, Chicago, IL 60611 USA

**Keywords:** Spinal, General, Anesthesia, Lumbar, Spine, Surgery, Delirium, Post-operative

## Abstract

**Background:**

Postoperative delirium is common in older adults, especially in those patients undergoing spine surgery, in whom it is estimated to occur in > 30% of patients. Although previously thought to be transient, it is now recognized that delirium is associated with both short- and long-term complications. Optimizing the depth of anesthesia may represent a modifiable strategy for delirium prevention. However, previous studies have generally not focused on reducing the depth of anesthesia beyond levels consistent with general anesthesia. Additionally, the results of prior studies have been conflicting. The primary aim of this study is to determine whether reduced depth of anesthesia using spinal anesthesia reduces the incidence of delirium after lumbar fusion surgery compared with general anesthesia.

**Methods:**

This single-center randomized controlled trial is enrolling 218 older adults undergoing lumbar fusion surgery. Patients are randomized to reduced depth of anesthesia in the context of spinal anesthesia with targeted sedation using processed electroencephalogram monitoring versus general anesthesia without processed electroencephalogram monitoring. All patients are evaluated for delirium using the Confusion Assessment Method for 3 days after surgery or until discharge and undergo assessments of cognition, function, health-related quality of life, and pain at 3- and 12-months after surgery. The primary outcome is any occurrence of delirium. The main secondary outcome is change in the Mini-Mental Status Examination (or telephone equivalent) at 3-months after surgery.

**Discussion:**

Delirium is an important complication after surgery in older adults. The results of this study will examine whether reduced depth of anesthesia using spinal anesthesia with targeted depth of sedation represents a modifiable intervention to reduce the incidence of delirium and other long-term outcomes. The results of this study will be presented at national meetings and published in peer-reviewed journals with the goal of improving perioperative outcomes for older adults.

**Trial registration:**

Clinicaltrials.gov, NCT03133845. This study was submitted to Clinicaltrials.gov on October 23, 2015; however, it was not formally registered until April 28, 2017 due to formatting requirements from the registry, so the formal registration is retrospective.

## Background

Post-operative delirium is a common occurrence in the elderly, with frequency estimates ranging from 10 to 50%, depending on the type of surgery [1]. For older adults undergoing spine surgery in particular, the incidence of delirium has been estimated to be > 30% [2]. This is a concern given that spine surgery is one of the top five procedures performed in this population [3]. Although previously thought to be transient with few long-term effects, it is now evident that delirium is associated with significant morbidity, including decreased functional status [4], increased duration of hospitalization [2, 5], increased mortality [6, 7], and cognitive decline [8–10]. In spite of the importance of postoperative delirium, there are few effective strategies to prevent delirium and long-term complications in older adults undergoing surgery.

Optimizing depth of anesthesia may represent a modifiable strategy for the prevention of delirium, with the hypothesis that excessive depth of anesthesia may have deleterious consequences for older adults. There are several potential mechanisms to support this hypothesis. In laboratory models, anesthetic agents have been shown to be neurotoxic, due to multiple mechanisms including enhanced neuronal apoptosis [11, 12], increased oligomerization and production of Alzheimer’s-associated amyloid-β protein [11, 12], and increased phosphorylation of Tau [13]. In clinical investigations, substantial anesthetic exposure may result in burst suppression on the electroencephalogram (EEG), and observational studies have shown an association between duration of burst suppression and mortality and delirium [14, 15], although a randomized trial showed no reduction in delirium from avoidance of burst suppression [16]. Finally, due to the hypotensive effects of anesthetic agents, EEG patterns consistent with deep anesthesia may in fact reflect inadequate cerebral blood flow [17].

In current practice, depth of anesthesia is determined by direct observation of hemodynamic and other autonomic responses to surgery, or with monitoring of the processed EEG (e.g., Bispectral Index [BIS], Medtronic, Inc., Andover, MA). The BIS monitor is a processed electroencephalogram which incorporates time-domain, frequency-domain, and bispectral analysis of raw EEG signals, and displays a dimensionless number ranging from 0 (isoelectric) to 100 (fully awake), with values < 60 consistent with general anesthesia [18, 19].

Prior to 2017, four randomized trials had demonstrated that using BIS to minimize the depth of anesthesia could reduce postoperative delirium [20–23]. Based on these trials, guidelines recommended that depth of anesthesia monitoring could be considered [24]. However, two recent trials using BIS or EEG-guidance during anesthesia failed to show a reductions in the frequency of delirium compared with standard care [16, 25]. Thus, the results of this body of literature are conflicting as to whether minimizing depth of anesthesia by monitoring processed EEG reduces the risk for postoperative delirium. Nonetheless, there are several points to consider in interpreting these data. First, in the majority of trials [16, 21–23], all patients received general anesthesia, and despite processed EEG monitoring, the intervention groups were maintained at a deep plane of anesthesia. Two other studies were conducted in patients undergoing spinal anesthesia for hip fracture surgery and randomization to “deep” vs. “light” sedation [20, 25]. However, the results were conflicting with one trial reporting a reduction in delirium in the “light” sedation group, but a follow-up study showing no difference. Of note, patients undergoing hip fracture have a high prevalence of dementia and 1-year mortality greater than 20% [26], and so results in this population would not be generalizable to most older adults undergoing surgery.

Thus, there is a clear need for further studies to determine whether reducing depth of anesthesia (beyond levels consistent with general anesthesia) can reduce delirium in a patient population that is generalizable to older adults whom are most susceptible to this complication. From a long-term perspective, it is unclear if preventing delirium may be a strategy to prevent cognitive and functional decline after hospitalization, and thus further study is needed to examine this question.

To address these gaps in understanding we are conducting the SHARP study, a prospectively randomized trial to determine whether reducing depth of anesthesia in the context of spinal anesthesia with targeted sedation versus general anesthesia can reduce the incidence of delirium after spine surgery in older adults. Spine surgery was chosen because of the high volume of the procedure in older adults and the associated high incidence of delirium. The study design is pragmatic since the interventions for each experimental group are bundled: the intervention arm receives spinal anesthesia with reduced depth of anesthesia [BIS targeted> 60–70] and propofol sedation, while the control arm receives general (deep) anesthesia with a volatile anesthetic. Because of the bundled interventions, depth of anesthesia will be linked with anesthetic technique. With regards to anesthetic technique, a large number of prior studies and meta-analyses have suggested that regional or neuraxial anesthesia does not reduce the incidence of delirium in comparison with general anesthesia [27, 28]. Similarly, there is no convincing evidence that intravenous anesthesia in comparison with volatile anesthesia reduces the incidence of delirium, although the quality of evidence is noted to be low [29, 30]. However, the depth of anesthesia was not accounted for in many of these studies, and is a key intervention in the current study, thus motivating the design and conduct of this trial. In the SHARP study, we will obtain preliminary data on whether a bundled intervention to reduce depth of anesthesia can improve cognition, functional status, disability, health-related quality of life, health care utilization, and pain at 3- and 12-months after surgery. In particular, there is some evidence that preemptive analgesia can reduce immediate postoperative analgesia requirements but long-term effects of pre-emptive analgesia are unclear, thus highlighting the importance of collecting pain and functional outcomes after discharge [31]. The primary hypothesis of the SHARP study is that reducing depth of anesthesia by using spinal anesthesia with targeted sedation will reduce the incidence of delirium after spine surgery in older adults compared with general anesthesia. The main secondary hypothesis is that the reduced depth of anesthesia group will have improvements in a screening measure of cognition compared to baseline.

## Methods/design

### Trial design and overview

**Fig. 1:**
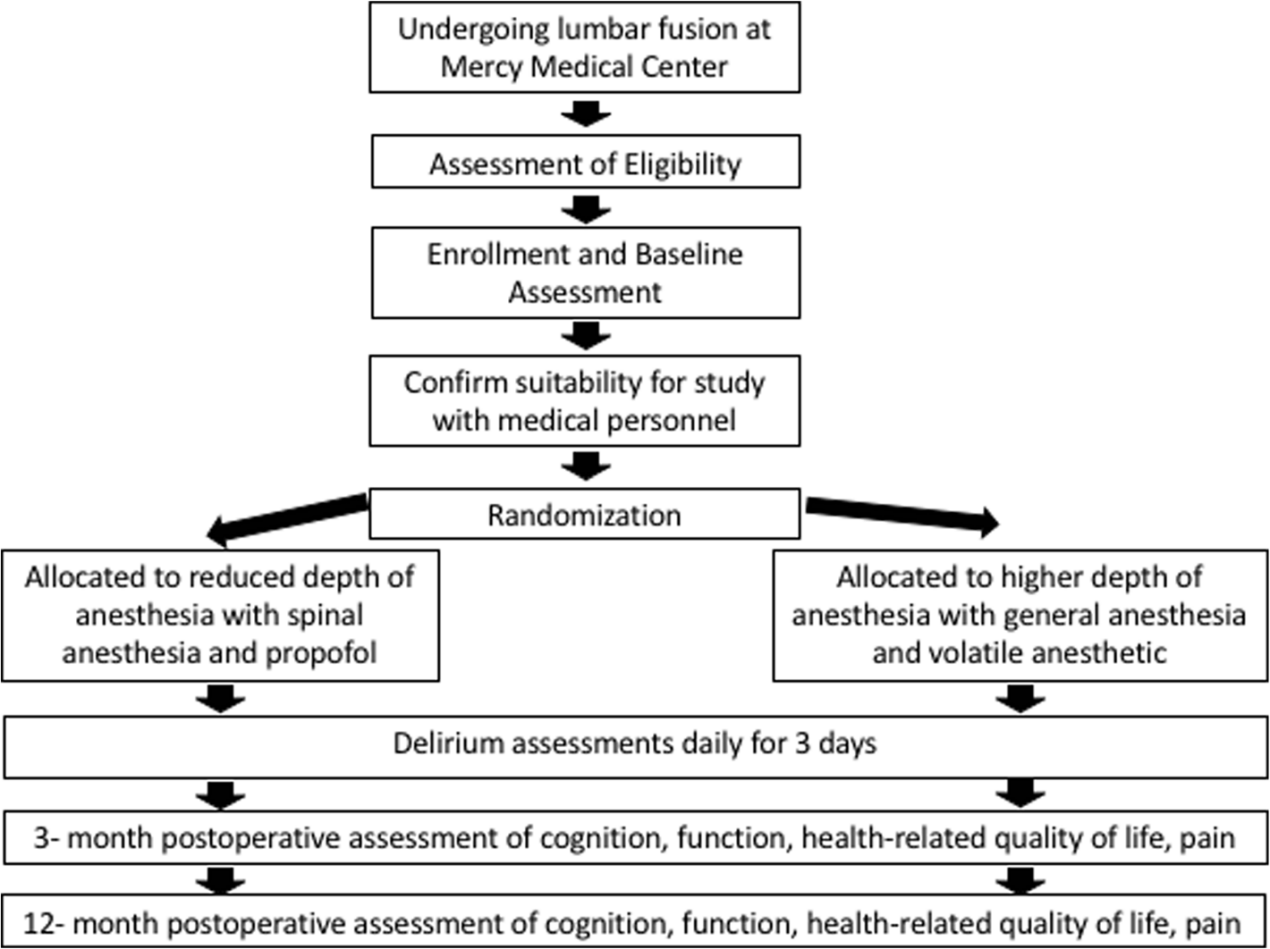
Study Flow Diagram. A flow diagram of study procedures is shown, including screening, enrollment, randomization, intervention, and follow-up assessments

The SHaping Anesthetic techniques to Reduce Postoperative delirium (SHARP) study is a single-center, prospective, randomized controlled superiority trial with two parallel groups. An overview of study procedures is shown in Fig. 1. The trial is enrolling patients 65 years and older undergoing lumbar spine fusion surgery. Participants are randomly assigned in a 1:1 allocation to spinal anesthesia with targeted intravenous sedation (intervention) or general anesthesia (control group). Assessments are conducted at baseline, in-hospital after surgery, and at 3- and 12- months after surgery. The primary outcome is the incidence of postoperative delirium. Secondary outcomes include the domains of cognition, function, disability, health-related quality of life, health care utilization, and pain.

### Study setting

The trial is being conducted at Mercy Medical Center in Baltimore, MD. The target completion date for study activities is June 2020.

### Eligibility criteria

Inclusion criteria for participation in the study are: 1) age 65 years or older, 2) undergoing a lumbar spine fusion surgery, 3) expected duration of surgery < 3 h, 4) under the care of a participating surgeon, and 5) expected ability to understand and comply with study procedures. Exclusion criteria are: 1) contraindications to spinal anesthesia (e.g. severe aortic stenosis, anti-coagulant or anti-platelet therapy), 2) BMI > 40, 3) prior L2–5 full lumbar fusion, 4) communication issues precluding baseline assessments, 5) baseline dementia or MMSE < 24, 6) psychiatric disease that would preclude cooperation with sedation with spinal anesthesia, and 7) attending surgeon or anesthesiologist preference for either spinal or general anesthesia for any reason due to clinical considerations.

### Patient recruitment

Research assistants screen patients for eligibility before scheduled surgery. Eligible patients are approached prior to surgery to discuss the study, and written informed consent is obtained prior to any study procedures by research staff. There are no financial incentives provided to patients. Before randomization, all clinicians (surgeons and anesthesiologists) are given the opportunity to withdraw the patient from the study for any reason, in particular if it is felt that the patient would clinically benefit from either general anesthesia or spinal anesthesia.

### Assignment of intervention

A computer-generated randomization list was created by a research nurse prior to initiation of the study. For the purpose of allocation concealment, patient assignments are placed in sealed opaque envelopes, which are sequentially opened by clinical staff after randomization, prior to entering the operating room.

### Intervention and control

The intervention group receives spinal anesthesia with targeted sedation, while the control group receives general anesthesia. To measure depth of anesthesia, all patients are monitored with a Bispectral Index (BIS) monitor. The BIS monitor is FDA-approved to monitor depth of anesthesia and displays a unit-less number (0–100) derived from processed EEG waveforms. BIS values between 40 and 60 are consistent with general anesthesia.

In the intervention group, spinal anesthesia is obtained through injection of 10–15 mg of bupivacaine into the subarachnoid space. Lidocaine may also be used for at the discretion of the attending anesthesiologist, based on the expected length of the surgical procedure. During the surgery, patients receive sedation with propofol (generally 25–150 mcg/kg/min), with guidance for sedation to be targeted to a BIS > 60–70. However, the anesthesiologist is instructed to prioritize any clinical concerns in the case that depth of sedation needs to be increased based on clinical judgment. In the event that the duration of the surgical procedure exceeds the analgesic effect of the spinal anesthetic, the surgeon will inject further intrathecal anesthetic under direct visualization, in consultation with the anesthesiologist.

In the control group, patients receive general anesthesia with an endotracheal tube. Guidelines are for anesthetic induction with propofol (generally 1–2 mg/kg) or etomidate, maintenance with a volatile anesthetic, muscle relaxation with a non-depolarizing muscle relaxant, and analgesia generally with fentanyl (generally 2–5 mcg/kg titrated). Hydromorphone and/or morphine is also acceptable. Patients on baseline opioids may receive additional opioids based on clinical criteria. Although patients under general anesthesia are also monitored with BIS, the anesthetic provider is masked to BIS values unless there is clinical need based on the individual anesthesiologist.

### Masking

Delirium outcome assessors are masked to the intervention. Postoperative data is abstracted from the electronic medical record by staff masked to the intervention. The assessments at 3- and 12- months are not masked. Throughout the study, patients, surgeons, and anesthesiologists are not masked, because it is virtually impossible for the anesthetic technique to be masked to treating physicians or patients. Statisticians and investigators involved in data analysis will be masked.

### Perioperative management

Perioperative care is according to clinical protocols at the Mercy Medical Center. Depending on the usual practice of the attending spine surgeon, patients may receive intrathecal opioids (generally 0.2–0.4 mg morphine) or local anesthetic. Patients receive fentanyl in the recovery room for pain. On the floor, patients use fentanyl or hydromorphone patient-controlled analgesia and transition to oxycodone or other oral opioids as tolerated.

### Data collection

Data is collected through patient interview and abstraction from the medical record. Patient medical and surgical history, preoperative medications, intraoperative medications and events, and postoperative medications, assessments, and events are abstracted from the medical record and stored in a Research Electronic Data Capture (REDCap) application. BIS data are downloaded and saved on a networked server at the end of each operation.

### Baseline and follow-up assessments

Patients undergo assessments prior to surgery and at 3- and 12-months postoperatively as described in Table 1. After surgery, research staff administer assessments in person at the 3-month clinical follow-up appointment, or by telephone in the case that follow-up assessments cannot be coordinated in person. The 12-month assessment is administered by telephone. Retention will be encouraged during each of these visits.

**Table 1:** Baseline and Follow-up Standardized Assessments

Neuropsychological Assessments	
Mini-Mental Status Examination ^a^	
Verbal Fluency Trials from the Calibrated Ideational Fluency Assessment	
Trail Making Test ^b^	
Digit Span Forwards/Backwards	
Function, Disability, and Health-Related Quality of Life Assessments	
Instrumental Activities of Daily Living	
Oswestry Disability Index	
12-Item Short Form Health Survey	
Pain scores	

^a^In the case of a telephone interview, the Telephone Interview for Cognitive Status is used

^b^The Trail Making Test is not administered in the case of telephone interview

### In-hospital delirium assessment

Delirium is assessed daily during the first three postoperative days using the Confusion Assessment Method (CAM) [32]. The CAM is a well-validated and standardized method for non-psychiatrists to diagnose delirium, with excellent sensitivity (94–100%) and specificity (90–95%). As part of the CAM assessment, trained study personnel visit the patient daily in the hospital and administer the following tests: Mini-Mental State Examination (MMSE) [33], Calendar Reverse Months, Shortened Digit Span Forward and Reverse, and Delayed Word Recall. Nursing staff, clinicians, and family and/or providers are also approached to provide further information for each patient assessment. Using this information, research staff complete the CAM algorithm. A chart review for delirium is also conducted using validated methods to supplement the in-person assessments [34]. Delirium severity is assessed using the Delirium Rating Scale-Revised 98 [35].

### Primary and secondary outcome

The primary outcome is incident delirium based on any positive Confusion Assessment Method (CAM). The primary secondary outcome is change in MMSE at 3-months after surgery.

### Other secondary outcomes

Additional domains that are important for postoperative recovery will be examined as secondary outcomes. These domains include: duration and severity of delirium, other tests of cognition, functional status, disability, health-related quality of life, health care utilization, and pain. The specific tests and outcomes for each of these domains are listed and defined in Table 2.

**Table 2:** Primary and Secondary Outcomes

Domain	Outcome	Definition
Delirium	Incident delirium *(Primary Outcome)*	Any CAM (+) assessment
	Delirium severity	Maximum DRS-R-98 score
	Number of days of delirium	Number of CAM (+) days
Cognition	Change in MMSE/TICS	Change in score. Item response theory will be used to compare similar or exact items if TICS> 50% of responses.
	Change in other individual test scores	Verbal Fluency (all trials combined, sum of scores), TMT-A (time), TMB-B - TMT-A (time), DSF (longest span correct), DSB (longest span correct)
Function	Any decline in IADL score	Any decline in IADL score from baseline to 3- and 12-month follow-up
Disability	Change in ODI score	Change in ODI score (%) from baseline to 3- and 12-month follow-up
Health-Related Quality of Life	Change in SF-12 PCS	Change in PCS score from baseline to 3- and 12-month follow-up
	Change in SF-12 MCS	Change in MCS score from baseline to 3- and 12-month follow-up
Health Care Utilization	Readmissions	% Readmissions
	Emergency department visits	% Emergency department visits
	Duration of hospitalization	Number of days in hospital after surgery
Pain	PACU pain	Last pain score in PACU.
		Total morphine equivalents
		Time to first opoid
	Hospital pain	Last pre-discharge pain score.
		Total morphine equivalents, pro-rated for 72 h in hospital
	Post-discharge pain	Average pain score at 3- and 12-month follow-up

Abbreviations: *CAM* Confusion Assessment Method, *DRS-R-98* Delirium Rating Scale-Revised 1998, *MMSE* Mini-Mental State Examination, *TMT* Trail Making Test, *DSF* Digit Span Forward, *DSB* Digit Span Backwards, *IADL* Instrumental Activities of Daily Living, *ODI* Oswestry Disability Index, *SF-12* 12-Item Short Form Health Survey, *PCS* physical component summary, *MCS* mental component summary, *PACU* postoperative anesthesia care unit

### Sample size

Assuming a delirium incidence of 35% in the control and a 50% reduction in the intervention group [2, 20], 206 patients would be needed to show a difference in incidence of delirium at a 0.05 significance level with a power of 0.8. With an estimated 6% withdrawal, the target enrollment is 218 patients. The expected sample size of 206 patients will also be sufficient for the secondary outcome of MMSE change at 3-months after surgery: the minimum detectable change in MMSE with 206 patients is ±0.235 at a 0.05 significance level with a power of 0.8.

### Data and statistical analysis

Data are entered on case report forms with subsequent entry into an on-line database application (REDCap). All paper forms are kept in a locked cabinet and are identified only with a study number. All REDCap databases are password protected and accessible only to authorized users. Data will be exported from REDCap to statistical package software in a SAFE environment for data analyses.

Data will first be evaluated for errors and patterns of missing data. Exploratory data analysis of patient characteristics and outcomes will be conducted, including visualization of the data using tables and graphs. Patients will be compared by group with respect to baseline characteristics collected for the cohort, including age, gender, race, education, comorbidities, medications, and perioperative characteristics, among others.

If the prevalence of missing data is < 5%, then primary analyses will be performed with the complete observed data. Sensitivity analyses will be performed using a best-worst case scenario, where all of those lost to follow-up in one group will be analyzed as having incident delirium and all of those lost to follow-up in the other group will be analyzed as not having incident delirium, and then vice versa; each of these results will then be qualitatively compared to the complete observed data results. If the prevalence of missing data is ≥5% but less than 40%, then multiple imputation methods will be employed; sensitivity analyses will compare the results from the multiple imputation analyses to the complete observed data analyses. If the prevalence of missing data is ≥40%, then analyses will be considered as preliminary and hypothesis-generating.

The primary analysis will be based on intention to treat principles, i.e. patients will be analyzed in the group to which they were randomized. For the primary outcome, incident delirium, both the absolute difference and relative change (i.e. relative incidence) will be computed, and the statistical test will be based on a two-sample test for proportions.

It is expected that < 10% of patients randomized to receive spinal anesthesia will actually receive general anesthesia due to failure to obtain spinal anesthesia. If patients receive both a spinal and general anesthetic, they will be categorized into an appropriate group for the on-treatment approach based on the biologic hypothesis for each analysis. For the purposes of the primary analysis on-treatment approach, they will be considered to have received deep anesthesia.

In a sensitivity analysis, we will consider accounting for crossover based on an inverse-probability of treatment weighting approach. This approach will provide an estimate of the difference in the incidence of delirium comparing the intervention and control groups, had all patients received their randomized treatment. Other sensitivity analyses will include: a) an as-treated analysis, b) re-estimation of both of the above treatment effects after adjustment for select baseline covariates, including age, education, and cognitive score [36], with consideration of gender, race, and other variables associated with incidence of delirium in bivariate analyses, and c) pre-specified subgroup analyses based on stratifying patients by age (< 75 vs. > 75 years old), baseline Charlson Comorbidity Index (0 vs. > 0), and baseline cognition (MMSE < 27 vs ≥27, or lowest quartile vs upper quartiles, dependent upon the baseline distribution). Confidence intervals and *p*-values will be generated via a bootstrap procedure using 5000 bootstrap samples.

The most important secondary outcome is change in MMSE (or Telephone Interview for Cognitive Status [TICS]) [37]. Change in MMSE/TICS will be analyzed using linear regression analysis, first unadjusted, and then with adjustment for age and education, with consideration of gender, race, and other variables associated with change in MMSE/TICS in bivariate analyses. If TICS is > 50% of responses, we will use item response theory to compare similar or exact items on the TICS and MMSE. The treatment effect will be defined as the difference in the change in MMSE/TICS at three months comparing the intervention and control groups. Other pre-specified secondary outcomes that will be compared between groups by domain are listed in Table 2 and include: (1) delirium severity and number of days of delirium, (2) change in each individual cognitive test score), (3) change in function, disability and quality of life (Instrumental Activities of Daily Living score [38] [any decline], Oswestry Disability Index score [39], Short Form (SF)-12 Physical Component Summary, SF-12 Mental Component Summary [40], (4) hospital utilization (readmissions, emergency department visits, duration of hospitalization, and (5) pain (pain in the postoperative anesthesia care unit, hospital pain, and post-discharge pain. The main post-discharge outcomes will be measured at 3-months. However, we will also collect and report data at 12-months after surgery. For continuous and binary secondary outcomes, similar methods as described above will be applied. We anticipate that delirium severity and number of days of delirium will be highly skewed and will be analyzed as ordinal or dichotomous variables.

Other pre-specified analyses include examining (1) the association of delirium and hospital utilization and other post-discharge outcomes (2) the association of BIS values and burst suppression with postoperative delirium and cognitive change, and (3) risk factors for decline in cognition, function, or health-related quality of life after spine surgery. In all analyses, a value of *p* < 0.05 will be considered significant. Due to the number of outcomes that will be examined (beyond incident delirium and change in MMSE), the analyses of secondary outcomes will be considered preliminary and hypothesis-generating.

### Strengths and limitations

The SHARP study has many important strengths. The trial is pragmatic and is conducted in a real-world clinical setting using standardized anesthetic approaches. The outcome of delirium is highly significant, and other outcomes are also important to both patients and clinicians. The intervention is not expensive and is able to be implemented by anesthesia providers using standard approaches. The investigative team is experienced in clinical studies in the perioperative setting using delirium as a primary outcome. The following limitations should be considered. The results of this single clinical trial in the spine surgery population would likely not be considered definitive, and subsequent studies would be needed to replicate any findings suggesting benefit to the intervention group (regardless of *p*-value). Some patients may not be appropriate for spinal anesthesia, and some anesthesia providers prefer general anesthesia with endotracheal intubation to secure the airway of patients in a prone patient. Thus, the results of this study may not be generalizable. The trial may be underpowered since a 50% reduction of delirium is assumed for the sample size estimation, and the estimate of 35% incidence of delirium may be too high in the control arm (with several large studies in non-cardiac surgery patients estimating an incidence of delirium in the 22–26% range [9, 16]. Nevertheless, a sample size of > 200 will provide important information. The intervention is a bundle of spinal anesthesia with targeted sedation with propofol, and thus if the intervention is effective, then further studies will be needed to understand precise mechanisms. Nevertheless, the trial design allows the comparison of “deep” general anesthesia with lighter levels of anesthesia than would be ethical to administer with general anesthesia. The trial design also reflects real-world clinical practice in anesthetic approaches. Outcome assessors at 3- and 12-months are not masked due to study logistical constraints. However, these assessments are highly standardized and the potential for bias is small. We also anticipate missing follow-up assessments at both 3- and 12-months due to patient retention in this cohort of older adults. As the MMSE cannot be administered over the telephone, we need to use another instrument to assess cognition for telephone follow-up. The TICS is the most widely used telephone instrument for assessment of global cognitive function, correlates well with the MMSE, and TICS scores and cutpoints have been directly linked to MMSE scores [41]. Finally, the MMSE has important limitations to consider, including practice and ceiling effects that may bias the results [42].

### Data monitoring

A Data Monitoring Safety Board (DSMB) has been established for oversight. The DSMB members are not direct participants in the study and they attest to not having any conflicts of interest. The DSMB is charged with oversight of the study’s safety and integrity and assessing the risk versus benefits of continuing the study if such questions arise. The DSMB meets regularly based on the progression of the study. The DSMB reviews patient recruitment and patient follow-up, compliance with the protocol including protocol violations, timeliness and completeness of data entry, compliance with patient confidentiality and HIPAA regulations, and communications of adverse events to the IRB. Members of the DSMB must maintain confidentiality of the study data until otherwise instructed. There is no provision for formal auditing beyond DSMB and IRB oversight, nor is there an interim analysis.

Data and safety for the study are primarily monitored by the principal investigators of the study. The principal investigators are informed of serious adverse events as soon as they occur for notification of the IRB and the Data Safety Monitoring Board (DSMB). The principal investigators (CB, CE) comprise the steering committee and developed the SHARP study protocol and are responsible for study oversight, protocol implementation, modification, and communication of changes, data analysis, and publication, and will have access to the final dataset. Protocol modifications are communicated to investigators, DSMB, and to trial registries through email and meetings.

The risks of this study derive from the clinical risks of the anesthetic care for the procedure. For this reason, the anesthesiologist and surgeon must agree that there is equipoise in the anesthetic choice prior to randomization. General risks for patients undergoing spinal anesthesia with sedation may include airway issues, patient movement, inadequate analgesia or anesthesia, neuraxial injury or hematoma, among others. General risks for patients undergoing general anesthesia include airway issues, oropharyngeal damage, and awareness, among others.

### Confidentiality

Only research staff have access to patient health information. Paper study forms are kept on file and stored in a locked cabinet in a locked office. Other electronic data are stored on password-protected department networked drives and are only accessible on hospital servers. REDCap is being used as the study database. A study ID is used to identify all participants.

### Dissemination

Results of this study will be presented at national meetings and published in peer-reviewed journals. Study data and analytic plan may be made available upon request to the principal investigators, with appropriate research and data-protection plans agreed upon.

## Discussion

As more older adults undergo surgery, the importance of preventing postoperative delirium and subsequent cognitive and functional changes has become paramount. In spine surgery in particular, improving postoperative pain is also an important consideration, especially for reducing the risk for delirium [2]. Optimizing the depth of anesthesia may be one strategy to reduce postoperative delirium, and the design of this pragmatic trial will test this hypothesis through a bundled intervention. The study was specifically designed to address a gap in the literature in which most trials examining the effects of depth of anesthesia have been conducted in patients undergoing general anesthesia. Thus, it is unclear whether an intervention to reduce anesthetic exposure to even lighter levels would be effective. To examine this question, we are employing a bundled intervention with spinal anesthesia and propofol titrated to a BIS greater than ~ 60–70, and we are using general anesthesia with a volatile anesthetic as the comparator group since this type of anesthetic choice is widespread. This study is pragmatic since each of these anesthetic choices are inter-twined but the resulting bundles of care are highly applicable to anesthesia practice.

Although the primary outcome of this study is postoperative delirium, secondary outcomes of cognition, function, health-related quality of life, and pain are also being measured at 3- and 12-months after surgery. This study will provide insight into whether reduced depth of anesthesia with spinal anesthesia might improve post-discharge outcomes, potentially through a mediating effect of delirium prevention. Additionally, the association of delirium and long-term outcomes, which has been examined in other populations, can also be extended into the spine surgery population.

We anticipate that the results of this study will provide important and generalizable information to improve the perioperative management of older adults undergoing spine surgery.

## Data Availability

Data sharing is not applicable to this article as no datasets were generated or analyzed during the current study.

## Abbreviations

BIS: Bispectral Index
CAM: Confusion Assessment Method
DRS-R-98: Delirium Rating Scale-Revised 1998
DSB: Digit Span Backwards
DSF: Digit Span Forward
DSMB: Data Monitoring Safety Board
EEG: Electroencephalogram
IADL: Instrumental Activities of Daily Living
IRB: Institutional Review
MCS: Mental component summary
MMSE: Mini-Mental State Examination
ODI: Oswestry Disability Index
PACU: Postoperative anesthesia care unit
PCS: Physical component summary
REDCap: Research Electronic Data Capture
SF: Short Form
SF-12: 12-Item Short Form Health Survey
SHARP: SHaping Anesthetic techniques to Reduce Postoperative delirium
TICS: Telephone Interview for Cognitive Status
TMT: Trail Making Test
